# Biomechanics of a calcar loading and a shortened tapered femoral stem: Comparative in-vitro testing of primary stability and strain distribution

**DOI:** 10.1186/s40634-021-00388-1

**Published:** 2021-09-07

**Authors:** Tobias Freitag, Ralf Bieger, Hartmuth Kiefer, Daniel Dornacher, Heiko Reichel, Anita Ignatius, Lutz Dürselen

**Affiliations:** 1 Department of Orthopaedic Surgery, Ulm University Medical Centre, Oberer Eselsberg 45, 89081 Ulm, Germany; 2 Department of Trauma and Orthopaedic Surgery, Lukas Hospital, Buende, Germany; 3 Institute of Orthopaedic Research and Biomechanics, Trauma Research Centre, Ulm University Medical Centre, Helmholtzstr. 14, 89081 Ulm, Germany

**Keywords:** Short stem, Primary stability, Stress-shielding, Total hip arthroplasty, Micromotions, Cadaver, Mechanical stress, Metaphyseal

## Abstract

**Purpose:**

The most common femoral short stems available on the market can, in principle, be divided with regard to their anchoring concepts into a calcar loading and a shortened tapered design. The purpose of this study was to compare the primary stability and stress-shielding of two short stems, which correspond to these two different anchoring concepts.

**Methods:**

Using seven paired fresh frozen human cadaver femurs, primary axial and rotational stabilities under dynamic load (100–1600 N) were evaluated by miniature displacement transducers after 100,000 load cycles. Changes in cortical strains were measured before and after implantation of both stem types to detect implant-specific load transmission and possible stress-shielding effects.

**Results:**

Reversible and irreversible micromotions under dynamic load displayed no significant differences between the two implants. Implantation of either stem types resulted in a reduction of cortical strains in the proximal femur, which was less pronounced for the calcar loading implant.

**Conclusions:**

Both short stems displayed comparable micromotions far below the critical threshold above which osseointegration may disturbed. Neither short stem could avoid proximal stress-shielding. This effect was less pronounced for the calcar loading short stem, which corresponds to a more physiological load transmission.

## Background

Despite the fact that there remains a lack of long-term data with respect to the expected survival of conventional implants, the number and market share of shorter femoral stems are increasing annually, with the anchoring concepts of this inhomogeneous implant group varying considerably [24]. The main objectives, which should be achieved by a shorter stem design, are to allow soft tissue and bone sparing implantation without negatively affecting implant survival [20]. In addition to abrasion-induced implant loosening, primary implant stability and stress-shielding are key determinants for implant durability [2, 8]. In-vitro measurements of primary implant stability under dynamic load and cortical strain distribution have the potential to predict clinical performance [2, 8]. Primary stability of cementless implants is determined on the one hand by reversible micromotions between the implant and the bone under dynamic loading and on the other hand by irreversible migration of the implant into the femoral canal in the sense of a settlement process [4]. The critical threshold for reversible micromotions, which enable successful osseointegration, was indicated as being 150 µm [23, 31]. According to this, implant-specific strain distribution and consecutive bone remodelling play an essential role for long-term implant survival [9, 28]. The metaphyseal anchorage concept of shorter femoral stems promises a more physiological load transfer with proximal strain distribution compared to conventional implants [3, 19]. Stress-shielding induces demineralisation of the proximal bone, resulting in compromised bone conditions that increase the risk for periprosthetic fractures [29] and complicate revision [36]. Results of the above-mentioned early biomechanical studies, which attempted to predict bone remodelling processes in the short- and mid-terms, were confirmed by recent clinical trials, which have shown reduced stress-shielding and consequently lower rates of proximal periprosthetic bone loss for several shorter femoral stems [16, 22, 25, 33]. However, the large variety of short stems with different anchoring philosophies inevitably leads to different bone remodelling patterns [32]. For this reason, it is of interest to examine each design with regard to its behaviour in the proximal femur. The variety of shorter femoral stems in principle can be categorised by their fixation philosophy and location of proximal loading. According to recently proposed classification systems [24], the most common short stems on the market can be assigned to type 2 (calcar loading) and type 4 (shortened tapered). Type 2 stems are intended to provide metaphyseal fixation through a wedge-shaped design in the frontal and sagittal planes within the cortical ring that remains after femoral neck osteotomy, whereas type 4 stems provide proximal fixation and force application typically through a rough proximal porous coating. In Germany, the type 2 concept of the Metha stem (Aesculap, Tuttlingen, Germany) and the type 4 concept of the Fitmore stem (Zimmer, Warsaw, Indiana) are currently among the most frequently used shorter femoral implants [18].

The aim of this biomechanical investigation was to determine whether there are differences between the two design concepts in terms of stress protection in the proximal femur and primary stability under dynamic loading. We hypothesised that both design concepts do achieve sufficient primary stability but not equally a proximal load transfer. To our knowledge, this is the first study to compare the biomechanical properties of these two implants.

## Methods

### Implants

**Fig. 1:**
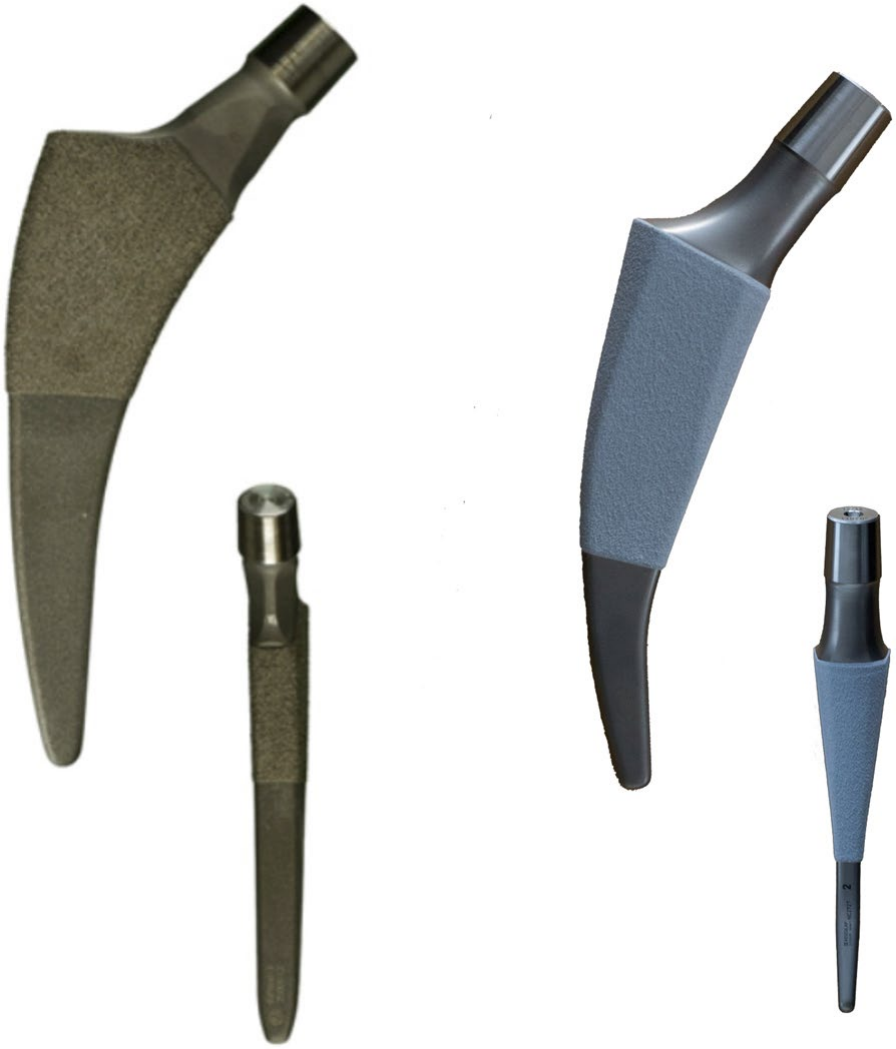
Anterior and sagittal profiles of the Fitmore (left) and the Metha stem (right)

Following institutional review board approval (No. 174/14), we investigated two types of femoral short stems made of titanium alloy with different anchoring concepts (Fig. 1). With the intention to enable reconstruction of the femoral offset, independent of the implant size, the Fitmore femoral stem is available with three different medial curvatures and one extended neck version. Femoral neck osteotomy is performed analogously to conventional straight stems. The primary press-fit is achieved by a triple conical design and a plasma-coating of the proximal third of the implant.

The Metha stem represents a calcar loading concept. Primary stability of this short stem is achieved by a wedge-shaped and triple-taper design with higher conicity in both the anteroposterior and the lateral planes, providing multiple-point contact. The stem also follows a metaphyseal anchoring concept with a primary fixation within the closed cortical ring of the femoral neck. This stem is available with three offset versions generated by the CCD-angle. Osseous integration is supported by a calcium phosphate coating of the entire proximal surface.

### Preparation of specimens

A total of seven paired fresh-frozen human femurs were received via ScienceCare (Phoenix, Arizona, USA). Six donors were male and one female, with a mean age of 54.1 years (range 28–61 years, Table 1). There were no malignant tumours or fractures in the donors’ history. Calibrated radiographs in two planes (anteroposterior, axial) of all femurs served as templates for implant planning and the exclusion of relevant deformities. Quantitative computed tomographies of each femur demonstrated no evidence of osteoporosis defined by a T-score of − 2.5 or lower (Table 1). Following basic preparation of the specimens, the femurs were resected to a length of 370 mm below the greater trochanter and fixed in a steel cup using methylmethacrylate (Technovit 3040; Heraeus Kulzer, Wehrheim, Germany) in a tilted position, laterally by 8° in the frontal plane and by 6° dorsally in the sagittal plane, to simulate a single leg stance and to create bending and torsional moments under load [1].

**Table 1:** Demographics of the donors (PS: Primary stability, CS: Cortical strain measurement)

Specimen	Side	Sex	Age	Height (m)	Weight (kg)	BMI (kg/m^2^)	T-Score	Implant	Size	Offset	Test
1	right	Male	59	1.78	95	30.1	0.3	Fitmore	6	B	PS
	left							Metha	2	135°	
2	left	Female	59	1.60	136	53.1	0.0	Metha	1	120°	PS
	right							Fitmore	6	B	
3	left	Male	55	1.83	88	26.3	0.3	Metha	3	130°	PS, CS
	right							Fitmore	9	B	
4	right	Male	58	1.85	136	39.7	1.3	Metha	2	130°	PS, CS
	left							Fitmore	9	B	
5	right	Male	59	1.73	101	33.9	− 0.7	Metha	3	130°	PS, CS
	left							Fitmore	9	B	
6	left	Male	28	1.88	137	38.6	0.0	Metha	3	130°	PS
	right							Fitmore	8	B	
7	right	Male	61	1.78	127	40.2	− 1.0	Metha	2	130°	PS
	left							Fitmore	10	B	
		Mean	54.1	1.8	117.1	37.4	0.0				
		SD	11.7	0.1	21.6	8.7	0.8				

### Measurement of reversible and irreversible micromotions under dynamic loading

**Fig. 2:**
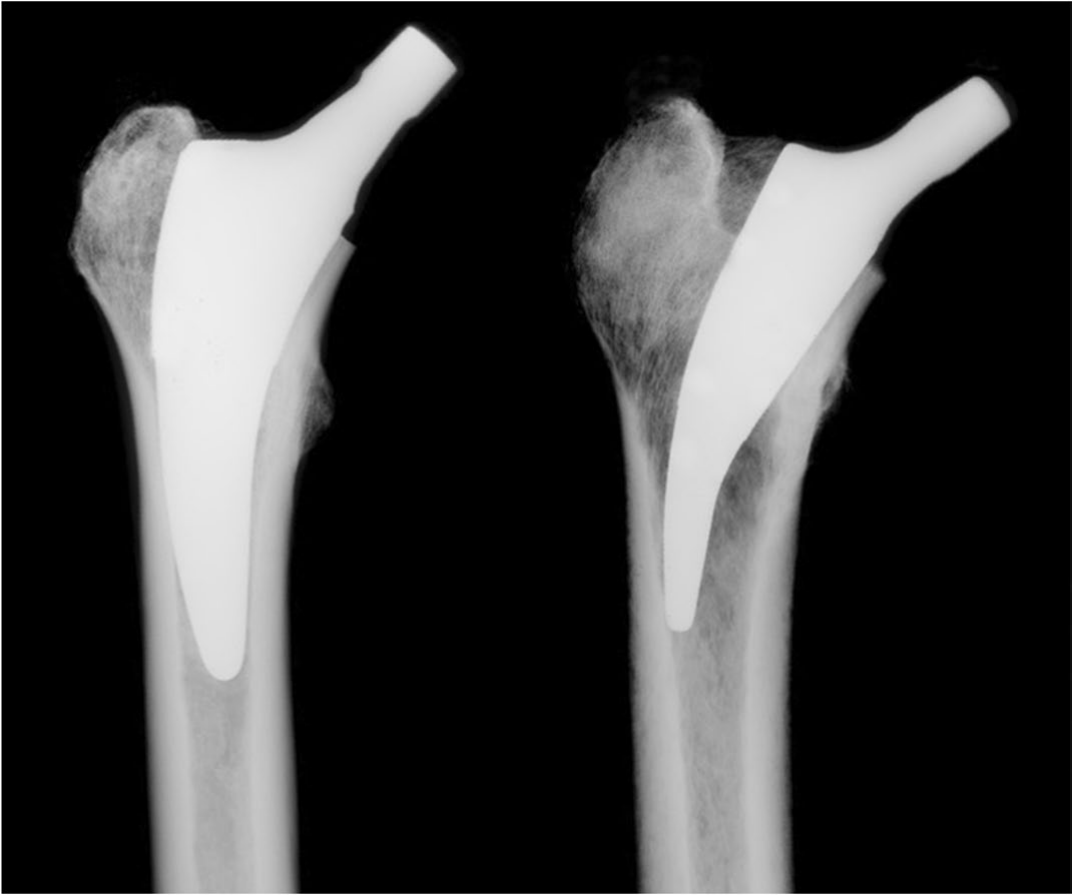
Radiographs of the Fitmore (left) and the Metha stem (right)

The test procedure has been described previously [2, 3]. The implantations of the short stems were performed alternating, either in the right or the left of the paired femurs, by an experienced orthopaedic surgeon according to the manufacturer's specifications (Fig. 2).

**Fig. 3:**
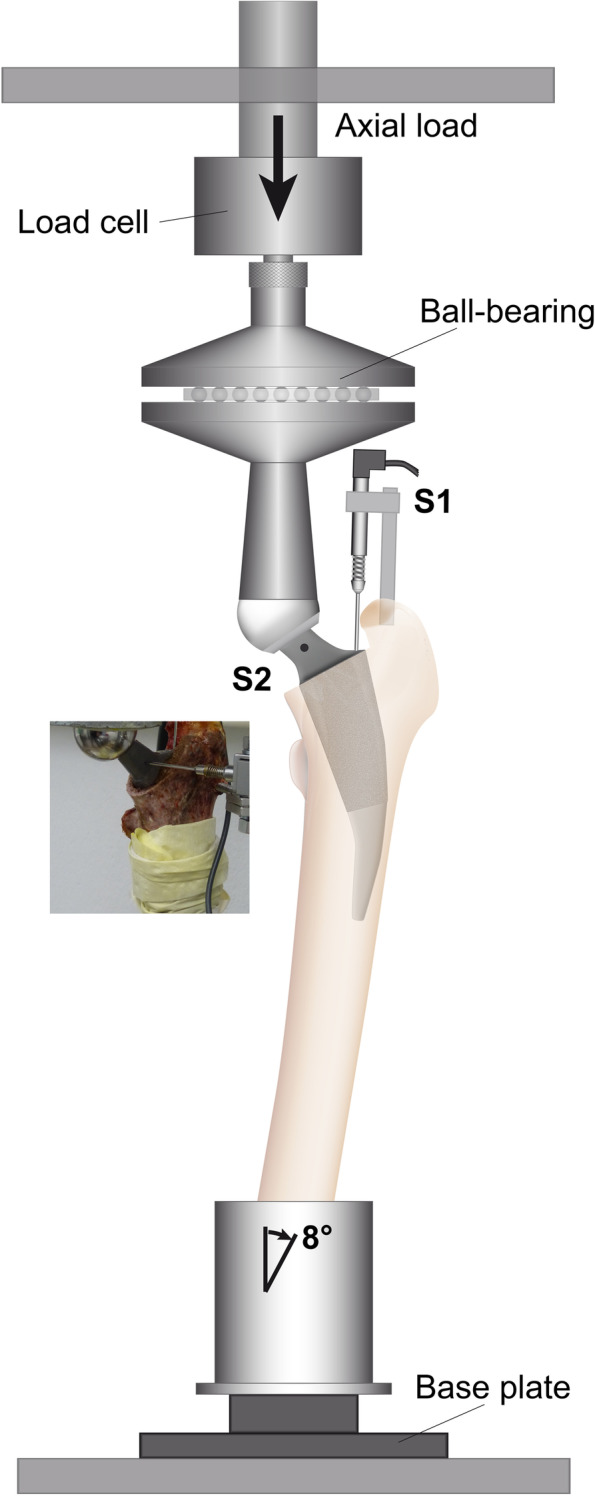
Illustration of the test set-up. S1 and S2 indicate the locations of the two miniature displacement transducers

Micromotion of the prostheses relative to the femoral cortex was measured with two inductive miniature displacement transducers (HBM WI/5 mm-T; HBM, Darmstadt, Germany) with an accuracy of 1 μm. The first transducer was attached to the greater trochanter with a 4.5 mm Schanz screw to detect axial micromotion and migration at the shoulder of the prosthesis (S1, Fig. 3). The tip of a second transducer was placed perpendicular to the neck of the prosthesis and was fixed with a Schanz screw to the greater trochanter (S2, Fig. 3). The measured micromotions were converted into rotation around the femoral axis by gauging the distance between the tip of the transducer and the longitudinal axis of the femoral diaphysis [2]. For dynamic loading, the femur was mounted in a servo hydraulic material testing machine (Instron, Typ 8871, Pfungstadt, Germany). Vertical load transmission through the prosthetic head (CoCr, 32 mm diameter) was transferred with a polyethylene acetabular cup. To create a moment-free contact, a ball-bearing plate was placed on top of the set-up (Fig. 3). The material testing machine applied 100,000 dynamic sinusoidal load cycles at a frequency of 2 Hz between 100 and 1600 N, which corresponds to approximately 2.5 times the bodyweight occurring during normal gait and to simulate the load of the first 6 weeks in vivo [1]. Reversible implant-bone motion was captured every 500 cycles at the two measurement points for all samples. For micromotion analysis, we used the mean amplitude of the final five cycles. Furthermore, irreversible implant migration in the axial direction (S1) was calculated by the displacement between the initial implant position and the position at the end of 100,000 loading cycles. In the same way, irreversible torsion around the femoral axis was calculated from the displacement assessed at transducer S2.

### Measurement of changes in cortical strains

To evaluate the stress-shielding behaviour of the implants, cortical strain measurements were performed on three of the seven pairs of human femurs before and after implantation of the prostheses. Each of the two prosthesis types was implanted alternatingly in one right and one left femur of each pair. For strain gauging, a previously described set-up was applied [2, 3].

**Fig. 4:**
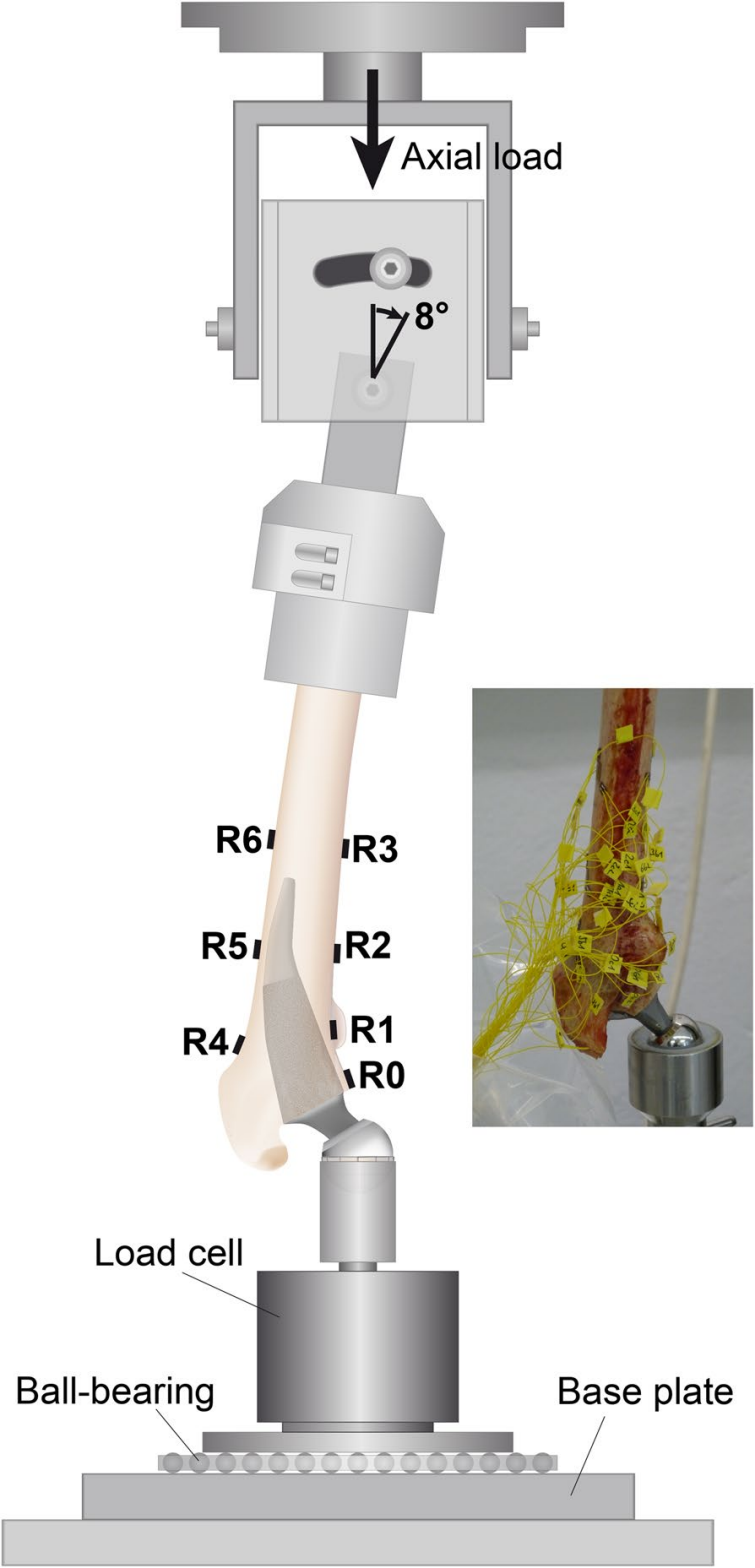
Illustration of the cortical strain measurement set-up. R0 to R6 show the positions of the strain gauge rosettes. R1 was attached medially at the level of the lesser trochanter and R4 laterally below the greater trochanter. The level of the middle section (R2 & R5) was 40 mm proximal and the distal rosettes (R3 & R6) were bonded 20 mm distal to the tip of the implant. R0 was attached medially 4 mm below the calcar osteotomy

For strain measurement, seven tri-axial strain-gauge rosettes (120 Ω, 6/120, RY11; HBM) were attached to the medial cortical surface and three laterally after templating the defined implant on X-ray images in two planes according to a standardised procedure [2, 3]. Two strain gauges were placed at the level of the trochanter minor (R1, R4, Fig. 4), two at 40 mm above the tip of the prosthesis (R2, R5) and two at 20 mm below the tip of the prosthesis (R3, R6). An additional strain gauge was placed 4 mm below the calcar osteotomy (R0). Finally, an unloaded rosette attached to the separated femoral condyles served as a temperature-compensating gauge. The strain gauge rosettes were oriented parallel to the femoral longitudinal axis. Strain gauge signals were recorded using a measurement amplifier (Canhead, HBM). The minimum and maximum principal strains were calculated from the signals measured by the three strain gauges of each rosette. The directions of these two strains were perpendicular to each other. In situations of compression, the absolute value was larger for the negative minimum principal strain than for the positive maximum principal strain, whereas in situations of tension, the positive maximum principal strain showed a larger absolute value. The strain with the larger absolute value was defined as the major strain.

The femur was fixed in an overhead position tilted laterally by 8° in the frontal plane as well as 6° dorsally in the sagittal plane. Three vertical loading cycles with a continuously increasing load each up to a maximum of 1600 N were applied with a material testing machine (Z010, Zwick GmbH, Ulm, Germany) to precondition the specimens and to determine repeatability (Fig. 4). Strains were measured for every load step during the third load application. After measuring the intact femur, the prosthesis was implanted and a neck length was selected to restore the original centre of rotation. To avoid experimental errors due to an altered head position after implantation and differences in the lever arm of the different femurs, results were calculated for a standardised bending moment of 53 Nm as previously described [7].

### Statistics

Statistical analysis was performed using SAS 9.4 software (SAS Institute, Cary, NC). Normality testing indicated that the data were non-parametric in nature, and testing was thus performed using Wilcoxon signed-rank to analyse differences of reversible and irreversible micromotions between the two implants. Significance was assumed for p ≤ 0.05. Because of the limited number of specimens used in the cortical strain measurement, the results were analysed descriptively.

## Results

### Reversible and irreversible micromotions under dynamic loading

The averaged amplitude of micromotions in the axial direction (S1) and the dynamic rotation around the central axis of the stem (S2) as well as the irreversible migration for both measurement points during the final five of the 100,000 loading cycles (1600 N) were far below the critical thresholds and almost identical for both stem models, with no statistical difference (Table 2).

**Table 2:** Measurements of reversible micromotion and irreversible migration after 100,000 loading cycles up to 1600 N

Implant	Mean	SD	Min	Max
Axial micromotion (S1) [µm] (*p* = 0.7)				
Fitmore	12	7	2	20
Metha	13	8	5	28
Rotational micromotion (S2) [°] (*p* = 0.4)				
Fitmore	0.06	0.02	0.02	0.09
Metha	0.05	0.06	0.01	0.17
Axial migration (S1) [µm] (*p* = 0.6)				
Fitmore	82	78	5	242
Metha	86	157	70	376
Rotational migration (S2) [°] (*p* = 0.4)				
Fitmore	0.2	0.2	0.05	0.37
Metha	0.3	0.3	0.10	0.73

### Changes in cortical strains

**Fig. 5:**
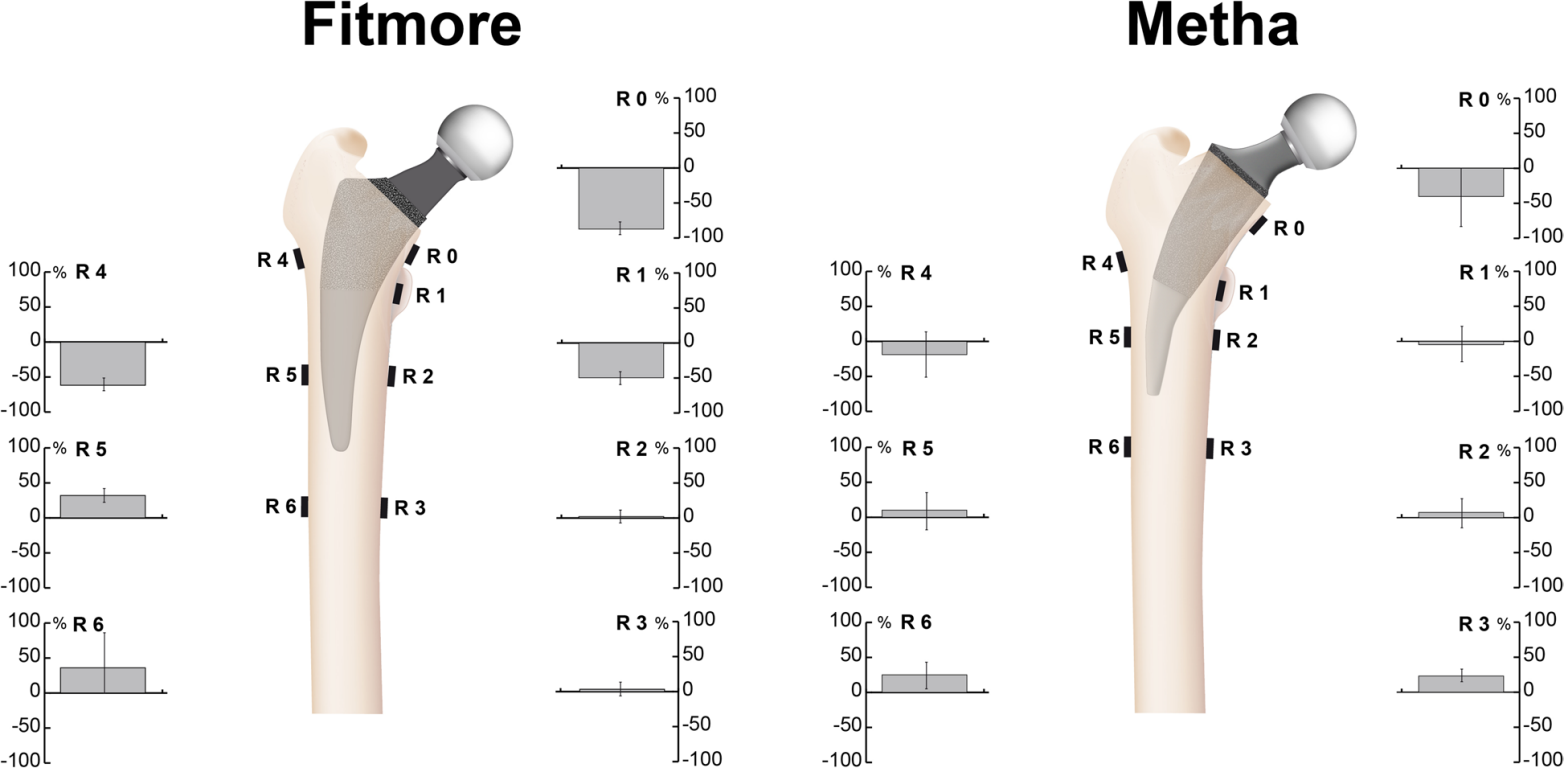
Mean percentage change in cortical surface strain after implantation of the Fitmore (left) and the Metha short stem (right) at the measurement positions R0–R6

The principal strains were compressive on the medial (R0–R3, Fig. 5) and tensile on the lateral side (R4–R6, Fig. 5) in the native bones as well as after implantation of the two prostheses. There was a tendency towards less stress reduction in the proximal femur (R0, R1, R4) after implantation of the Metha short stem (Fig. 5). In the middle and distal sections (R2, R3 and R5, R6), mean changes showed only minor differences between the two stems. The mean measurements at positions R0 (Metha: − 40.2% ± 59.5%, Fitmore: − 87.2% ± 9.1%), R1 (Metha: − 4.7% ± 27.4%, Fitmore: − 49.6% ± 8.9%) and R4 (Metha: − 18.8% ± 44.0%, Fitmore: − 61.7% ± 8.3%) indicated a reduction in the surface strain for both implants. At positions R2 (Metha: 7.4% ± 21.6%, Fitmore: 2.0% ± 10.9%), R3 (Metha: 23.1% ± 8.9%, Fitmore: 3.5% ± 13.4%), R5 (Metha: 10.2% ± 29.4%, Fitmore: 32% ± 14.8%) and R6 (Metha: 25.1% ± 21.6%, Fitmore: 36% ± 45%) an increased surface strain was observed after implantation of both hip stems.

## Discussion

The aim of this study was to compare the primary stability and strain distribution of a calcar loading (Metha) and a shortened tapered stem (Fitmore). We found a high and almost equivalent primary stability for both stem designs. Furthermore, our results demonstrated reduced proximal stress-shielding with a tendency to more physiological load transfer for the Metha stem, although a stress-shielding effect was present for both stems. The results are in agreement with our hypothesis.

That a shorter stem design, in endoprosthetic treatments of the hip with good bone quality, does not negatively affect primary stability is well examined in biomechanical studies [2, 3, 32]. However, it must be noted that results of different in-vitro studies are not reliably comparable due to different test protocols and test preparations [17]. For this reason, comparative studies are necessary, particularly to minimise the impact of anatomical variations [6].

Due to the metaphyseal anchorage concept of shorter stem designs, the comparison with a conventional stem model has shown a more favourable rotational stability for a type 2 and a type 4 short stem in two separate studies with an almost identical test-setup [2, 3]. Similar results of the in-vitro primary stability were obtained in a study comparing two type 2 short stems with a thrust plate prosthesis, for which good long-term data already existed [14]. In this three-dimensional test setup using composite femora and a load application of up to 1700 N, reversible micromotions at all measuring points were likewise less than 150 µm. This is also supported by available excellent clinical mid-term results of these implant designs [13, 21, 34]. Yamako et al. reported controversial observations of an experimental study, which examined shorter variants of an anatomical stem [37]. This in-vitro study using composite bones with a similar maximum loading of 1600 N showed increasing micromotions in the axial and rotational directions for shortened variants of the conventional model (120 mm) by 40 mm and 70 mm, respectively. This investigation indicated that simply shortening a conventional implant may not necessarily achieve the required stability. Multipoint cortical fixation, particularly within the cortical neck level, supported by cancellous bone compaction of the type 2 stem, as well as the tapered-wedge design with a large coronary diameter of the proximal implant third of the type 4 stem are design features that already have shown sufficient primary stability in earlier in-vitro studies [2, 3, 14, 30].

The measured proximal stress reduction of the Fitmore stem corresponds to previous measurements of this stem model of our research group [2]. In this in-vitro study, the comparison with a type 2 stem (Mayo, Zimmer, Warsaw, Indiana) showed no significant difference in strain distribution. By contrast, the comparison with a conventional straight stem (CLS Spotorno, Zimmer, Warsaw, Indiana) showed a more physiological load transfer for the Fitmore stem. This was also supported by clinical trials measuring periprosthetic bone density changes [16, 27]. Interestingly, Maier et al. reported a high rate of postoperative cortical hypertrophy mainly in zones 3 and 5 according to Gruen for this implant model in a single centre study without negatively affecting clinical outcome [26]. The authors proposed a reduced primary stability for this phenomenon. However, these hypotheses were countered by an associated, comparative biomechanical in-vitro study using synthetic bones and a dynamic axial load of up to 4000 N with a proven conventional straight stem [30]. Primary stability in the present study did not support these hypotheses either. Another hypothesis for this observation would be a more distal load transmission and consequent bone remodelling. However, findings of a dual-energy X-ray absorptiometry (DEXA) study over 5 years of this stem model counter this consideration [27]. In this investigation, the Fitmore stem showed no corresponding change in bone mineral density (BMD) of the distal third of the implant and less proximal stress-shielding compared to the CLS straight stem. The current study also showed no relevant difference in the change of the cortical surface strain in this area compared to the Metha stem. For both the CLS and the Metha stem used as references in these studies, no frequent occurrence of cortical hypertrophies was described. However, the stress-shielding effect around the proximal third of the implant was less pronounced for the Metha stem in the present study.

A biomechanical study using synthetic bones showed that the resection height appears to have a considerable influence on the load transmission of the Metha stem in the proximal femur [12]. The deeper the resection, the more physiological were the strain patterns in this investigation. This aspect was not considered in the present study. However, in this implant group, the resection height directly determines the stem position, so that this effect is directly related to the individual anatomy to be reconstructed. Furthermore, a lower resection height negatively influences the rotational stability of a femoral implant [35]. In contrast to these findings, a finite element model investigating the design-specific effect of stress-shielding of shorter femoral stems depending on the resection height showed no difference in behaviour in the proximal femur [5]. In our study, a preferably calcar-sparing resection height was chosen according to the preoperative planning of the implant position in reconstructing individual anatomy.

The influence of the offset variant used on load transmission in the proximal femur represents another essential aspect. A biomechanical comparison of different offset variants of a modular conventional straight stem demonstrated only minor changes on overall femoral load transmission [10], whereas varus implant positioning resulted in a significant increase in the distal load transfer [15]. Relevant changes in the proximal third of the implant, particularly in the calcar region, were not found in this biomechanical investigation. However, a transfer of these results to shorter femoral stems appears to be limited. For the Metha stem with a small CCD-angle and correspondingly larger offset reconstruction, a higher load transmission in the calcar region and only minor changes in the distal third were observed in a biomechanical study using synthetic bones [11]. In the present study, both implants displayed a decrease in the cortical surface strain of the calcar region, which was less pronounced for the Metha stem. We used the offset variant according to the preoperative planning, taking into consideration the native femoral offset. However, in the present study, a high offset stem variant was used with only one specimen and not included for the cortical strain measurement. Furthermore, an increased load transmission in the calcar region by implantation of a high offset stem in a usually normal configured synthetic bone can be expected and probably does not correspond to the situation in vivo. Corresponding to our results, a biomechanical study using synthetic bones and a Metha stem with a CCD-angle of 135° demonstrated a reduction of the cortical surface strain in the calcar region [19]. Furthermore, this is in agreement with findings of a DXA study involving 25 patients after total hip arthroplasty (THA) with a Metha stem, which showed a decrease of BMD in the region of the calcar of approximately 13% after two years [25].

This study has some limitations. First and importantly, this experimental model was intended to simulate conditions that occur during the first postoperative weeks and might underestimate individual activity levels. Torsional and bending moments were generated by simulating a single-leg stand and a load application, which corresponds to normal gait and weight. We are aware that in-vitro studies are only partially able to reflect conditions in vivo. In particular, the influence of muscle force on implant behaviour in the femoral bone was not considered in this test setup. Nonetheless, this offers the advantage of high reproducibility [4, 7]. We only used strain gauges positioned in the frontal plane. Particularly in the case of a pronounced implant tilt in the sagittal plane, which can occur with a high osteotomy and native femoral torsion, load transmission of the distal implant third may be underestimated by the chosen strain gauge positions. We are aware that gauge position R0 depends on the osteotomy level and, therefore, the measurement results are not directly comparable. Nevertheless, we have chosen this positioning to gain information at the level of the calcar osteotomy, which could best detect a proximal load introduction. Cortical strain measurement does not take into consideration adaptive bone remodelling. Nevertheless, clinical studies investigating changes in bone mineral density after THA are consistent with in-vitro findings for several stem designs [16, 22, 25]. Nonetheless, regarding statistical analyses, the power of the analyses is restricted by the small number of cases. For this reason, only a descriptive analysis was made for cortical strain measurements.

In conclusion, the current study demonstrated that both short stems achieved high primary stability, with micromotions well below the critical threshold above which osseointegration may be disturbed. Both stems could not avoid proximal stress-shielding. The design concept of the Metha stem with a comparatively large proximal rectangular cross-section, which provides for a cortical implant contact at the osteotomy level, ensures a more favourable load transmission with regard to the effect of stress-shielding. Clinical studies need to evaluate whether the design concepts differ in terms of the long-term performance.

## Data Availability

The data that support the findings of this study are available from the corresponding author (TF), upon reasonable request.

## References

[1] Bergmann G, Graichen F, Rohlmann A (1993). Hip joint loading during walking and running, measured in two patients. J Biomech.

[2] Bieger R, Ignatius A, Decking R, Claes L, Reichel H, Durselen L (2012). Primary stability and strain distribution of cementless hip stems as a function of implant design. Clin Biomech.

[3] Bieger R, Ignatius A, Reichel H, Durselen L (2013). Biomechanics of a short stem: In vitro primary stability and stress shielding of a conservative cementless hip stem. J Orthop Res.

[4] Buhler DW, Oxland TR, Nolte LP (1997). Design and evaluation of a device for measuring three-dimensional micromotions of press-fit femoral stem prostheses. Med Eng Phys.

[5] Burchard R, Braas S, Soost C, Graw JA, Schmitt J (2017). Bone preserving level of osteotomy in short-stem total hip arthroplasty does not influence stress shielding dimensions - a comparing finite elements analysis. BMC Musculoskelet Disord.

[6] Claes L, Fiedler S, Ohnmacht M, Duda GN (2000). Initial stability of fully and partially cemented femoral stems. Clin Biomech.

[7] Cristofolini L (1997). A critical analysis of stress shielding evaluation of hip prostheses. Crit Rev Biomed Eng.

[8] Decking R, Puhl W, Simon U, Claes LE (2006). Changes in strain distribution of loaded proximal femora caused by different types of cementless femoral stems. Clin Biomech.

[9] Engh CA, Bobyn JD, Glassman AH (1987). Porous-coated hip replacement. The factors governing bone ingrowth, stress shielding, and clinical results. J Bone Joint Surg Br.

[10] Enoksen CH, Gjerdet NR, Klaksvik J, Arthursson AJ, Schnell-Husby O, Wik TS (2016). Deformation pattern and load transfer of an uncemented femoral stem with modular necks. An experimental study in human cadaver femurs. Clin Biomech.

[11] Floerkemeier T, Budde S, Hurschler C, Lewinski G, Windhagen H, Gronewold J (2017). Influence of size and CCD-angle of a short stem hip arthroplasty on strain patterns of the proximal femur - an experimental study. Acta Bioeng Biomech.

[12] Floerkemeier T, Gronewold J, Berner S (2013). The influence of resection height on proximal femoral strain patterns after Metha short stem hip arthroplasty: an experimental study on composite femora. Int Orthop.

[13] Floerkemeier T, Tscheuschner N, Calliess T (2012). Cementless short stem hip arthroplasty METHA(R) as an encouraging option in adults with osteonecrosis of the femoral head. Arch Orthop Trauma Surg.

[14] Fottner A, Schmid M, Birkenmaier C, Mazoochian F, Plitz W, Volkmar J (2009). Biomechanical evaluation of two types of short-stemmed hip prostheses compared to the trust plate prosthesis by three-dimensional measurement of micromotions. Clin Biomech.

[15] Fottner A, Woiczinski M, Kistler M (2018). Varus malalignment of cementless hip stems provides sufficient primary stability but highly increases distal strain distribution. Clin Biomech.

[16] Freitag T, Hein MA, Wernerus D, Reichel H, Bieger R (2016). Bone remodelling after femoral short stem implantation in total hip arthroplasty: 1-year results from a randomized DEXA study. Arch Orthop Trauma Surg.

[17] Gheduzzi S, Miles AW (2007). A review of pre-clinical testing of femoral stem subsidence and comparison with clinical data. Proc Inst Mech Eng H.

[18] Grimberg A, Jansson V, Lützner J, Melsheimer O, Morlock M, Steinbrück A (2020). Endoprothesenregister Deutschland (EPRD)—Jahresbericht 2020. EPRD; Berlin, Germany

[19] Gronewold J, Berner S, Olender G (2014). Changes in strain patterns after implantation of a short stem with metaphyseal anchorage compared to a standard stem: an experimental study in synthetic bone. Orthop Rev.

[20] Huo MH, Parvizi J, Bal BS, Mont MA (2009). What’s new in total hip arthroplasty. J Bone Joint Surg Am.

[21] Innmann MM, Weishorn J, Bruckner T (2019). Fifty-six percent of proximal femoral cortical hypertrophies 6 to 10 years after total hip arthroplasty with a short cementless curved hip stem - a cause for concern?. BMC Musculoskelet Disord.

[22] Jahnke A, Engl S, Altmeyer C (2014). Changes of periprosthetic bone density after a cementless short hip stem: a clinical and radiological analysis. Int Orthop.

[23] Jasty M, Bragdon C, Burke D, O’Connor D, Lowenstein J, Harris WH (1997). In vivo skeletal responses to porous-surfaced implants subjected to small induced motions. J Bone Joint Surg Am.

[24] Khanuja HS, Banerjee S, Jain D, Pivec R, Mont MA (2014). Short bone-conserving stems in cementless hip arthroplasty. J Bone Joint Surg Am.

[25] Lerch M, Kurtz A, Stukenborg-Colsman C (2012). Bone remodeling after total hip arthroplasty with a short stemmed metaphyseal loading implant: finite element analysis validated by a prospective DEXA investigation. J Orthop Res.

[26] Maier MW, Streit MR, Innmann MM (2015). Cortical hypertrophy with a short, curved uncemented hip stem does not have any clinical impact during early follow-up. BMC Musculoskelet Disord.

[27] Meyer JS, Freitag T, Reichel H, Bieger R (2019). Periprosthetic bone mineral density changes after implantation of a curved bone preserving hip stem compared to a standard length straight stem: 5-yr results of a prospective, randomized DXA-analysis. J Clin Densitom.

[28] Oh I, Harris WH (1978). Proximal strain distribution in the loaded femur. An in vitro comparison of the distributions in the intact femur and after insertion of different hip-replacement femoral components. J Bone Joint Surg Am.

[29] Peitgen DS, Innmann MM, Merle C, Gotterbarm T, Moradi B, Streit MR (2018). Periprosthetic bone mineral density around uncemented titanium stems in the second and third decade after total hip arthroplasty: A DXA study after 12, 17 and 21 years. Calcif Tissue Int.

[30] Pepke W, Nadorf J, Ewerbeck V (2014). Primary stability of the fitmore stem: biomechanical comparison. Int Orthop.

[31] Pilliar RM, Lee JM, Maniatopoulos C (1986) Observations on the effect of movement on bone ingrowth into porous-surfaced implants. Clin Orthop Relat Res 208(2):108–113 3720113

[32] Small SR, Hensley SE, Cook PL (2017). Characterization of femoral component initial stability and cortical strain in a reduced stem-length design. J Arthroplasty.

[33] Synder M, Krajewski K, Sibinski M, Drobniewski M (2015). Periprosthetic bone remodeling around short stem. Orthopedics.

[34] von Lewinski G, Floerkemeier T (2015). 10-year experience with short stem total hip arthroplasty. Orthopedics.

[35] Whiteside LA, White SE, McCarthy DS (1995). Effect of neck resection on torsional stability of cementless total hip replacement. Am J Orthop.

[36] Wilkinson JM, Hamer AJ, Rogers A, Stockley I, Eastell R (2003). Bone mineral density and biochemical markers of bone turnover in aseptic loosening after total hip arthroplasty. J Orthop Res.

[37] Yamako G, Chosa E, Totoribe K, Watanabe S, Sakamoto T (2015). Trade-off between stress shielding and initial stability on an anatomical cementless stem shortening: in-vitro biomechanical study. Med Eng Phys.

